# The Demographic and Clinical Characteristics of Oral Squamous Cell Carcinoma: An Institutional Epidemiological Study

**DOI:** 10.7759/cureus.82552

**Published:** 2025-04-18

**Authors:** Deivanayagi M, Ramesh Thanikachalam, Narmadha C, Elamparithi B, Akila K, Adhithya Baskaran

**Affiliations:** 1 Oral Medicine and Radiology, Adhiparasakthi Dental College and Hospital, Melmaruvathur, IND; 2 General Medicine, Melmaruvathur Adhiparasakthi Institute of Medical Sciences, Tamil Nadu, India, Melmaruvathur, IND; 3 Dentistry, Adhiparasakthi Dental College and Hospital, Melmaruvathur, IND; 4 Oral Pathology, Adhiparasakthi Dental College and Hospital, Chennai, IND

**Keywords:** oral cancer, oral squamous cell carcinoma, prevalence, tobacco, tongue

## Abstract

Background

Oral squamous cell carcinoma (OSCC), a malignant epithelial neoplasm, is a major health concern in the Indian subcontinent, ranking among the top three cancers. With increasing reports of OSCC affecting younger individuals and variations in site-specific prevalence, targeted epidemiological research is crucial. The primary objective of this study was to evaluate the demographic and clinical prevalence of OSCC in a rural South Indian population.

Materials and methods

This retrospective study was conducted in the Department of Oral Medicine and Radiology at Adhiparasakthi Dental College and Hospital, Melmaruvathur. The study analyzed 90 histopathologically confirmed OSCC cases from 2021 to 2023. Data was collected on patient demographics, specific oral cavity sites affected, and etiological factors, including tobacco and alcohol use, and statistically analyzed using descriptive statistics and the Chi-squared test.

Results

Among the 90 participants, 51.1% were males and 48.9% were females. Most cases, 55 (61.1%), were aged between 51 and 75 years. The left buccal mucosa was the most common site for OSCC, accounting for 35.6% of cases. This was followed by the right buccal mucosa with 18.9%, and the lateral border of the tongue with 14.4%. Etiologically, 42% of participants used only smokeless tobacco, and 12% used both smoke and smokeless tobacco. Moderately differentiated OSCC was the most prevalent histological type, accounting for 52.2% of cases.

Conclusion

The study highlights a significant prevalence of OSCC in rural areas, emphasizing the need for targeted preventive measures, education, early detection, and improved healthcare access to reduce the disease burden in these regions. The findings also suggest a potential link between site-specific tobacco placement and lesion location, meriting further investigation.

## Introduction

Oral cancer is one of the most commonly occurring cancers worldwide. In 1977, Pindborg et al. defined OSCC as "a malignant epithelial neoplasm exhibiting squamous differentiation as characterized by the formation of keratin and/or the presence of intercellular bridges" [1]. Oral squamous cell carcinoma is a major health concern in the Indian subcontinent, consistently ranking among the top three most prevalent cancers in the region [2]. It typically arises from the mucosal lining of the oral cavity, including the palate, lips, gingiva, tongue, and retromolar trigone.

Tobacco is a primary cause of oral cancer, and the association between various cancers and specific smokeless tobacco (SLT) products is influenced by the type of product, method of use, and inherent toxicity. Chewing tobacco, in particular, has been linked to a significantly higher risk of oral and oesophageal cancers. The carcinogenic potential of SLT is attributed to the ingestion and metabolic activation of harmful compounds present in these products, which initiate and promote carcinogenesis [3]. In addition to tobacco, poor oral hygiene, alcohol consumption, nutritional deficiencies, low socioeconomic status, and chronic trauma from dental appliances or sharp teeth play an etiological role [4].

OSCC most commonly develops on the tongue. This is due to its exposure to various carcinogens from tobacco and alcohol consumption, as well as potential trauma from sharp teeth or dental appliances [5]. Addressing the rising trend of OSCC in young individuals necessitates a comprehensive approach involving education, prevention, and early detection strategies [6]. In rural areas of middle-income countries, healthcare infrastructure is often underdeveloped, with fewer trained healthcare providers and limited access to essential health services, including cancer screening, diagnosis, and treatment facilities. As a result, cancer cases in rural areas are frequently diagnosed at advanced stages. Addressing these disparities requires comprehensive strategies aimed at improving healthcare infrastructure, training healthcare providers, implementing effective cancer screening programs, and increasing awareness and education about cancer prevention and early detection in rural communities [7].

Early detection through regular screenings and lifestyle changes can significantly improve outcomes. Early diagnosis plays a crucial role in cancer treatment outcomes. It not only enables timely and effective treatment but also reduces the physical, psychological, and financial burden on the patient and their families. It may improve the survival rate up to 90% [8]. The population where a high incidence of oral cancer was observed predominantly came from rural areas. This was attributed to their low socioeconomic status, casual attitude towards health, high tobacco consumption, and limited access to healthcare facilities [7]. Therefore, this study aims to assess the demographic and clinical profile of patients diagnosed with OSCC in a rural Indian population, thereby contributing to the understanding of the disease burden and its etiological factors. The specific objectives are to analyze the age and gender distribution of OSCC, determine the most frequently affected anatomical sites, evaluate the influence of tobacco and alcohol use, and explore the correlation between duration of habits and cancer occurrence. By identifying these patterns, this study seeks to inform targeted public health interventions and reinforce the need for community-based screening and education programs aimed at reducing the incidence and improving early diagnosis of OSCC in underserved areas.

## Materials and methods

This retrospective study was conducted at Adhiparasakthi Dental College and Hospital, Melmaruvathur, in the Department of Oral Medicine and Radiology. The study obtained ethical clearance from the Institutional Review Board (2024/IRB-FEB-39/APDCH). The study analyzed parameters such as age, gender, specific anatomical sites of the oral cavity, and etiological factors associated with OSCC. 

The sample size for this retrospective study was determined based on the availability of complete histopathologically confirmed cases of oral squamous cell carcinoma (OSCC) within the study period from 2021 to 2023. A total of 90 patient records that met the inclusion criteria were selected for analysis. Data were collected from the archives of the Department of Oral Medicine and Radiology and the Department of Oral Pathology between the years 2021 and 2023. Subjects were categorized by gender into male and female groups, and the age range of participants was between 18 and 80 years. The age group was further divided into ages 18-25, 26-50, 51-75, and 76- 80.

The distribution of OSCC cases was documented based on anatomical locations, including the buccal mucosa, retromolar trigone, floor of the oral cavity, tongue, gingivo-buccal sulcus, palate (both hard and soft palate), maxillary and mandibular alveolus, upper lip, and lower lip. Additionally, the study considered the history of tobacco consumption and alcohol intake, detailing the frequency and duration of these habits. Habit history was retrieved from the case record archives. The cases lacking histopathological records are excluded from the study.

Statistical analysis

Descriptive statistics were used to summarize demographic and clinical characteristics. Categorical variables were analyzed using the Chi-squared test to determine the association between gender, anatomical site, and etiological factors. The level of statistical significance was set at p<0.05. All statistical analyses were performed using SPSS version 20 (IBM Inc., Armonk, New York).

## Results

Demographic characteristics

**Figure 1:**
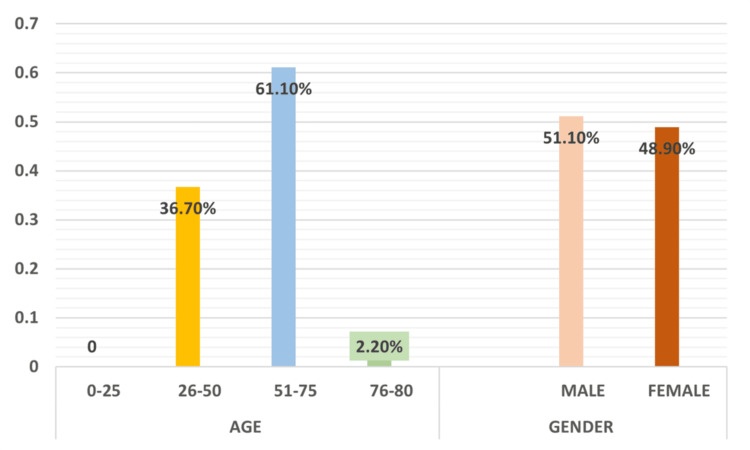
Distribution of study samples based on age and gender

The study included data from 90 participants to assess the demographic and clinical prevalence of OSCC. The distribution of the population based on gender revealed that 51.1% of participants were males, while 48.9% were females. Regarding age groups, the majority of participants (61.1%) were between 51 and 75 years old. Participants aged 26 to 50 years constituted 36.7% of the study population, whereas those aged 76 to 80 years made up 2.2%, as illustrated in Figure 1.

Etiological factors

**Figure 2:**
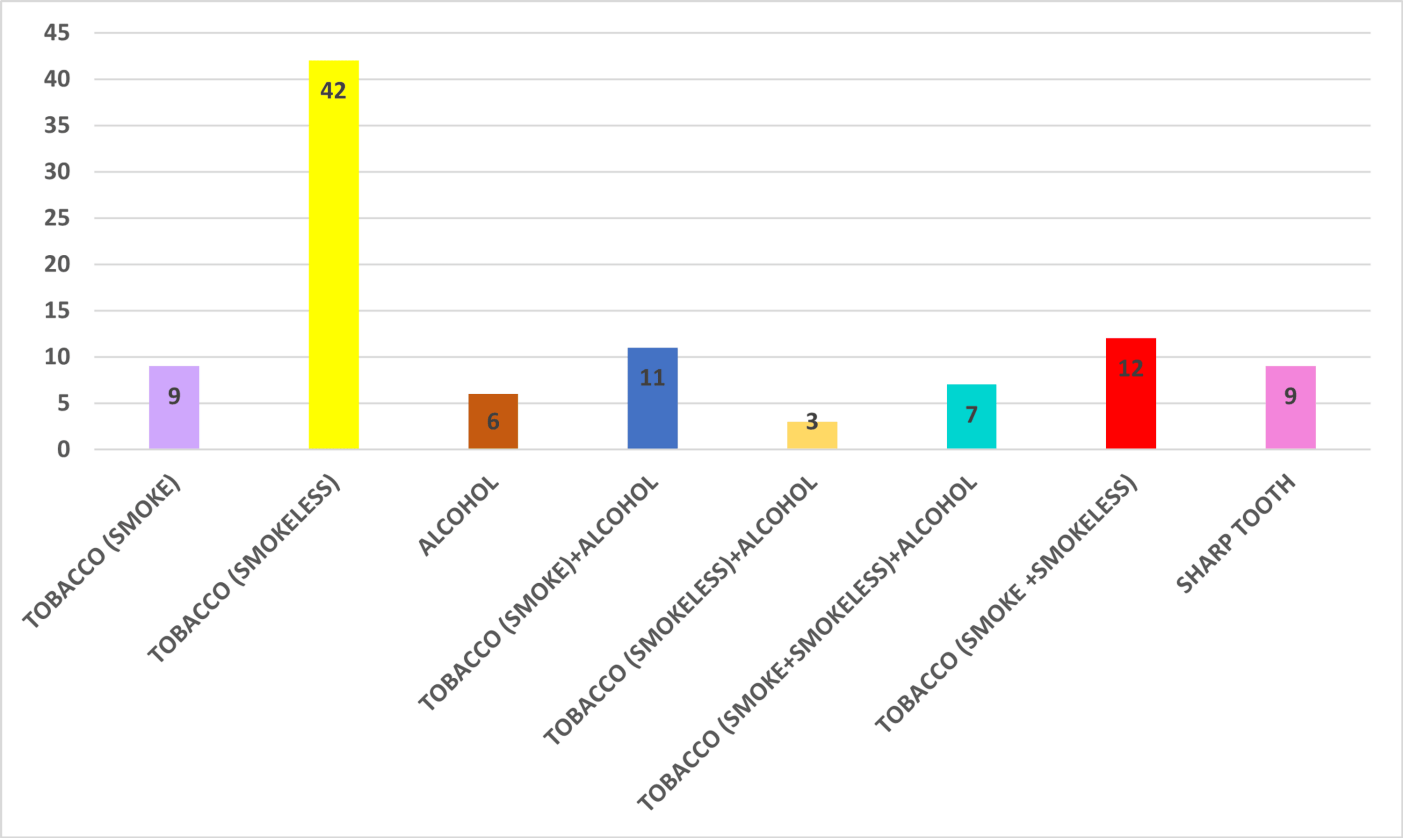
Distribution of etiological factors among the study population

Figure 2 illustrates the analysis of the distribution of different etiological factors among the study population. The study sample showed that 42% of the patients used smokeless tobacco. Table 1 illustrates the study's findings on the duration of tobacco usage and the occurrence of cancer lesions among participants.

Anatomical site distribution

On analysis of site distribution, Table 1 shows that the left buccal mucosa is the most prevalent site for oral squamous cell carcinoma, representing 35.6% of cases.

**Table 1:** Distribution of oral squamous cell carcinoma on various sites

Site	Frequency	Percentage
Left buccal mucosa	32	35.6
Right buccal mucosa	17	18.9
Lateral border of the tongue	13	14.4
Right mandibular alveolus	8	8.9
Left mandibular alveolus	7	7.8
Floor of the mouth	4	4.4
Left maxillary alveolus	3	3.3
Right maxillary alveolus	3	3.3
Left gingivo buccal sulcus	3	3.3

Table 2 represents the duration of tobacco usage and the occurrence of cancer lesions. The Chi-squared test results showed no significant relationship between age and carcinoma type (p=0.176) or between gender and carcinoma type (p=0.185). Specifically, the differentiation levels of well, moderate, and poor showed no statistically significant relationship with age or gender in the study.

**Table 2:** Distribution of duration of tobacco usage and occurrence of cancer lesion among the study population

Duration of tobacco use	Frequency of cancer occurrence	Percentage of cancer occurrence
0-5 years	18	20.0
5-10 years	23	25.6
10-15 years	22	24.4
>20 years	27	30.0

Histological differentiation

Table 3 depicts how demographic details and carcinoma types were analyzed for their associations within the study population.

**Table 3:** Relationship between age, gender, and carcinoma type among the study population

Parameter	Options	Carcinoma type differentiation levels			Chi-squared	sig
		Well	Moderate	Poor		
Age	18-25 years	0	0	0	6.333	0.176
	26-50 years	11	18	4		
	51-75 years	24	28	3		
	76-80 years	0	1	1		
Gender	Male	21	23	2	3.379	0.185
	Female	14	24	6		

Table 4 depicts how demographic details and etiological factors were analyzed for their associations within the study population. Significant statistical associations were found in gender and duration comparisons (p-value=0.000), indicating that gender and duration of usage significantly influence the development of OSCC. Conversely, age did not demonstrate any significant association with etiological factors, suggesting that age is not a determining factor in this population's progression to oral squamous cell carcinoma. These findings underscore the importance of considering gender and duration of usage when assessing the risk factors associated with this type of cancer.

**Table 4:** Relationship between demographic details, types and duration of habits among the study population

Demographic details	Etiological factors								sig
	Smoking	Smokeless	Alcohol	Smoking + alcohol	Smokeless + alcohol	Smoking + smokeless +alcohol	Smoking + smokeless	Sharp tooth	
Age 18-25 years	0	0	0	0	0	0	0	0	0.895
Age 26-50 years	5	16	2	4	1	1	4	-	
Age 51-75 years	4	25	4	6	2	6	8	-	
AGE 76-80 years	0	1	0	1	0	0	0	-	
Male	7	9	6	11	3	3	7	0	0.000
Female	2	33	0	0	0	4	5	0	
0-5 years of tobacco usage	1	2	0	2	0	1	12	0	0.000
5-10 years of tobacco usage	1	14	1	4	0	3	0	0	
10-15 years of tobacco usage	2	14	2	1	2	1	0	0	
>20 years of tobacco usage	5	12	3	4	1	2	0	0	

## Discussion

This study aimed to document the prevalence of oral cancer in a rural population over five years from 2021 to 2023 based on hospital reports. We examined the variation in the occurrence of oral squamous cell carcinoma across different anatomical sites of the oral cavity, including its etiology, duration, commonly affected age groups and genders, and the type of carcinoma. Out of 90 OSCC cases analyzed, the majority were aged 51-75 years, with a nearly equal gender distribution. The left buccal mucosa was the most commonly affected site (35.6%). Smokeless tobacco was the predominant etiological factor (46.7%), followed by mixed habits. Moderately differentiated OSCC was the most prevalent histological type (52.2%). Park et al. (2010) reported a higher proportion of early-stage diagnoses (60.8%) in the young group compared to 37 patients (59.7%) in the older group [9]. This is in accordance with our study.

Conversely, a higher percentage of advanced-stage diagnoses, 40.3%, were noted in the older individuals versus 39.2% in the younger individuals. These contrasting diagnostic stages between age groups underscore the complex nature of OSCC across different age brackets, warranting further investigation. Our current research supports this finding, revealing a significant occurrence of OSCC among individuals aged 51 to 75 years.

Several studies based on the Surveillance, Epidemiology, and End Results Program (SEER) database in the USA have highlighted a rising incidence of oral cancer among younger individuals [10]. Tandon et al. in 2017 found that oral squamous cell carcinoma most frequently affects the buccal mucosa. They also noted that the mandibular alveolus was the second most affected location, with the tongue being the third in the Rural Health Care Centre in Maharashtra [11]. In a study by Shenoi et al. (2012), out of 295 individuals diagnosed with squamous cell carcinoma, the gender distribution noted that 238 patients (81%) were male, and 57 patients (19%) were female [12]. However, the gender distribution in our study appears more balanced (51.1% male vs. 48.9% female).

Chamoli et al. (2021) recently conducted a study on OSCC in the South Indian population, highlighting a notable prevalence among individuals aged 40 and above compared to younger age groups [13]. According to Dwivedi et al. (2023), their study found that smokeless tobacco, predominantly in the form of gutka, was the most prevalent, followed by smoked tobacco such as bidi [14]. Our study data similarly indicate smokeless tobacco as the most common, followed by both smoked and smokeless forms. Excessive tobacco use is responsible for 1 in 10 deaths worldwide and over 5 million fatalities annually. Smokeless tobacco alone contributes to approximately 42% of oral cancer cases, primarily due to the substantial release of reactive oxygen species during chewing. Tobacco-specific nitrosamines and reactive oxygen species are crucial in triggering OSCC associated with chewing tobacco [15].

People who use both alcohol and tobacco face a significantly increased risk of developing OSCC because of the combined effect of these substances. Alcohol dehydrates cell membranes, making oral tissues more permeable to the carcinogens found in tobacco [16]. Pyne et al. (2018), after analysing a total of 1666 SCCs, reported a distribution of 1,367 cases (82.1%) of well-differentiated SCCs, 222 cases (13.3%) of moderately differentiated SCCs, and 77 cases (4.6%) of poorly differentiated SCCs [17]. On analysing the literature, a notable difference was found in the prevalence of well-differentiated SCCs in Sydney, Australia. However, in our study, the well-differentiated OSCC was higher (38.9%). These findings underscore variations in the histopathological profiles of OSCC, highlighting the importance of comprehensive data analysis for understanding disease trends and outcomes.

Singh et al. (2020) reported higher OSCC incidence in patients aged ≥31 years, with well-differentiated types common in older age groups. Similarly, our study found most OSCC cases in patients aged 51-75 years across all differentiation types. Poorly differentiated OSCC was less common and rarer in younger individuals in both studies. These findings highlight a consistent age-related pattern in OSCC differentiation. Age stratification remains crucial for understanding OSCC behaviour [18].

This retrospective, single-institution study may be prone to selection and recall bias, limiting the robustness of its findings. The reliance on archival data introduced challenges due to incomplete or inconsistent case records. Generalizability is also limited due to the localized study setting. These factors collectively highlight the need for broader, multicentric prospective studies in the future.

## Conclusions

This retrospective study examined 90 cases of oral squamous cell carcinoma (OSCC) and found a higher prevalence among individuals aged 51-75 years, with the left buccal mucosa being the most commonly affected site. Smokeless tobacco use was identified as the predominant etiological factor, and moderately differentiated OSCC was the most prevalent histological type. A statistically significant association was noted between gender and the duration of tobacco usage, underscoring behavioral influence in disease development. While these findings provide insight into the demographic and clinical patterns of OSCC in a rural setting, the study's single-institution design and retrospective nature limit generalizability. Further multicentric and prospective research incorporating broader etiological data and clinical staging is essential to strengthen understanding and inform targeted preventive strategies. 
